# Supplementary material for: Uptake of Radionuclides by Bryophytes in the Chornobyl Exclusion Zone
Source: Toxics. 2023 Feb 25;11(3):218. doi: 10.3390/toxics11030218 (PMC10056093; doi:10.3390/toxics11030218)
Supplement: Supplementary file 1 [file toxics-11-00218-s001.zip › toxics-2172463-supplementary.pdf]

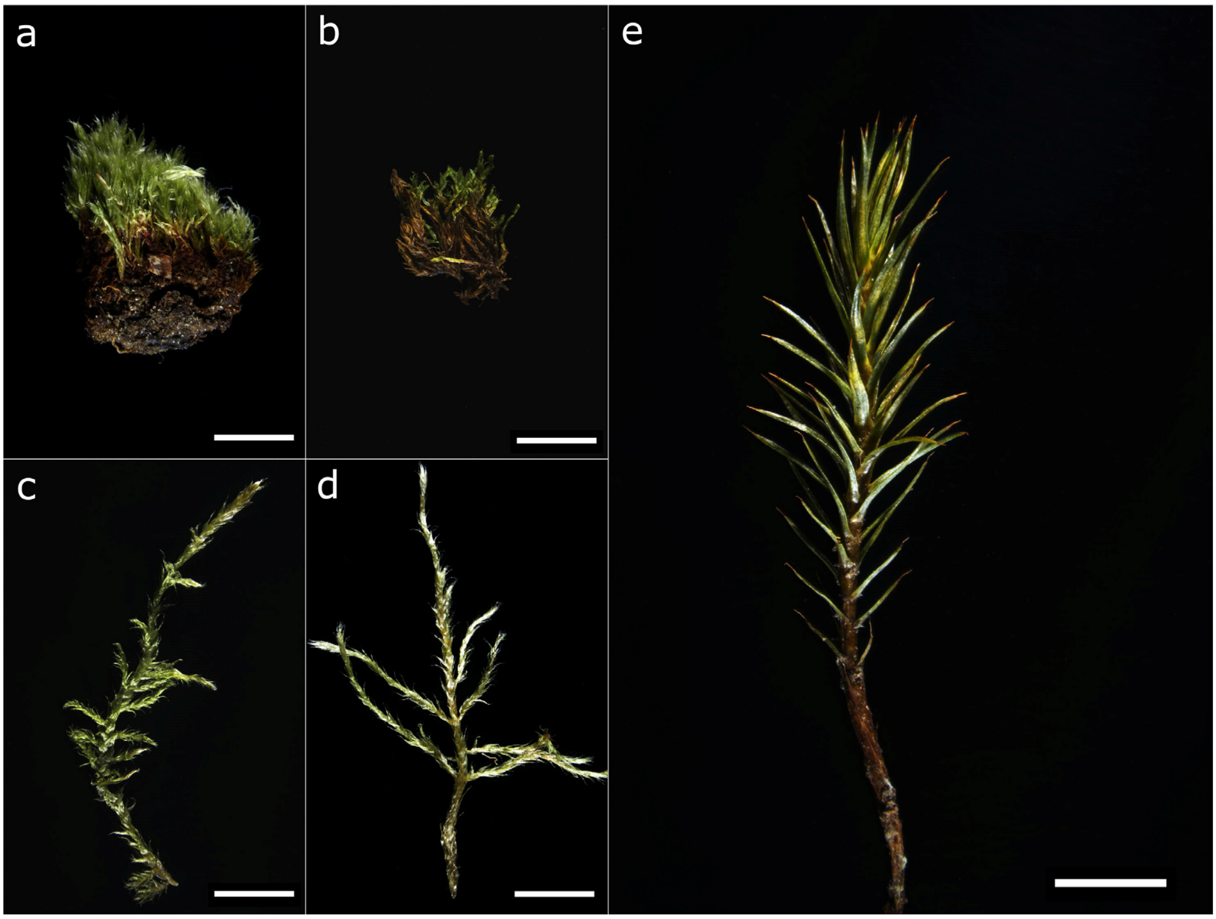

Figure S1. Additional figure of sampled mosses. # indicate sampling sites after Figure 1. (**a**) *Bryum cf. badium* (#12), (**b**) *Ceratodon purpureus* (#3), (**c**) *Brachythecium glareosum* (#15), (**d**) *Amblystegium serpens* #20 (**e**) *Polytrichum juniperinum* (#22). Scalebar = 5 mm

Table S1. Results of Kruskal-Wallis tests and regression analyses. Correlation coefficients are only shown if the regression model contains one co-founder.

**Isotope**	**Independent Variable**	**P Value (KW Test)**	**F Value (Regression Model)**	**R^2 (Regression)**	**P Value (Regression)**	**Correlation Coefficient (Regression)**
^{137}Cs	Bryaceae vs. Other	0.001	0.002	0.34	n.a.	n.a.
	Taxonomic Group	0.062	0.134	0.17	n.a.	n.a.
	Substrate	0.029	0.002	0.83	n.a.	n.a.
	Conductive Tissue Y/N	0.174	0.756	0.004	0.76	−70.9 (−591–431)
	Distance to Unit 4	n.a.	< 0.001	0.445	< 0.001	470.4 (241–700)
	Fallout 1986 (90Sr)	0.423	0.523	0.018	0.52	400.8 (−890–1701)
^{90}Sr	Bryaceae vs. Other	0.439	0.151	0.36	n.a.	n.a.
	Taxonomic Group	0.617	0.659	0.04	n.a.	n.a.
	Substrate	0.206	< 0.001	1	n.a.	n.a.
	Conductive Tissue Y/N	0.617	0.659	0.042	0.66	20.4 (−101–150)
	Distance to Unit 4	n.a.	0.432	0.127	0.43	−0.8 (−30.3–10.6)
	Fallout 1986 (90Sr)	n.a.	n.a.	n.a.	n.a.	n.a.
^{241}Am	Bryaceae vs. Other	0.944	0.52	0.02	n.a.	n.a.
	Taxonomic Group	0.223	0.239	0.12	n.a.	n.a.
	Substrate	0.019	0.009	0.78	n.a.	n.a.
	Conductive Tissue Y/N	0.066	0.033	0.182	0.03	0.1 (0–0.2)
	Distance to Unit 4	n.a.	0.761	0.004	0.76	0 (−0.1–0)

Fallout 1986 (90Sr)	0.152	0.379	0.034	0.38	−0.1 (−0.3–0.1)
